# Evaluation of the systemic and mucosal immune response induced by COVID-19 and the BNT162b2 mRNA vaccine for SARS-CoV-2

**DOI:** 10.1371/journal.pone.0263861

**Published:** 2022-10-18

**Authors:** Olaf Nickel, Alexandra Rockstroh, Johannes Wolf, Susann Landgraf, Sven Kalbitz, Nils Kellner, Michael Borte, Corinna Pietsch, Jasmin Fertey, Christoph Lübbert, Sebastian Ulbert, Stephan Borte

**Affiliations:** 1 Department of Laboratory Medicine, Hospital St. Georg, Leipzig, Germany; 2 Fraunhofer Institute for Cell Therapy and Immunology, Leipzig, Germany; 3 Fraunhofer Cluster of Excellence Immune-Mediated Diseases CIMD, Leipzig, Germany; 4 ImmunoDeficiencyCenter Leipzig, Jeffrey Modell Diagnostic and Research Center for Primary Immunodeficiency Diseases, Hospital St. Georg, Leipzig, Germany; 5 Hospital Vaccination Center, Hospital St. Georg, Leipzig, Germany; 6 Department of Infectious Diseases/Tropical Medicine, Nephrology and Rheumatology, Hospital St. Georg, Leipzig, Germany; 7 Institute of Medical Microbiology and Virology, Leipzig University Hospital, Leipzig, Germany; 8 Interdisciplinary Center for Infectious Diseases, Leipzig University Hospital, Leipzig, Germany; 9 Division of Infectious Diseases and Tropical Medicine, Department of Medicine II, Leipzig University Hospital, Leipzig, Germany; 10 Division of Clinical Immunology and Transfusion Medicine, Department of Laboratory Medicine, Karolinska Institutet, Karolinska University Hospital Huddinge, Stockholm, Sweden; Qatar University, QATAR

## Abstract

**Background:**

The currently used SARS-CoV-2 mRNA vaccines have proven to induce a strong and protective immune response. However, functional relevance of vaccine-generated antibodies and their temporal progression are still poorly understood. Thus, the central aim of this study is to gain a better understanding of systemic and mucosal humoral immune response after mRNA vaccination with BNT162b2.

**Methods:**

We compared antibody production against the S1 subunit and the RBD of the SARS-CoV-2 spike protein in sera of BNT162b2 vaccinees, heterologous ChAdOx1-S/BNT162b2 vaccinees and COVID-19 patients. We monitored the neutralizing humoral response against SARS-CoV-2 wildtype strain and three VOCs over a period of up to eight months after second and after a subsequent third vaccination.

**Results:**

In comparison to COVID-19 patients, vaccinees showed higher or similar amounts of S1- and RBD-binding antibodies but similar or lower virus neutralizing titers. Antibodies peaked two weeks after the second dose, followed by a decrease three and eight months later. Neutralizing antibodies (nAbs) poorly correlated with S1-IgG levels but strongly with RBD-IgGAM titers. After second vaccination we observed a reduced vaccine-induced neutralizing capacity against VOCs, especially against the Omicron variant. Compared to the nAb levels after the second vaccination, the neutralizing capacity against wildtype strain and VOCs was significantly enhanced after third vaccination. In saliva samples, relevant levels of RBD antibodies were detected in convalescent samples but not in vaccinees.

**Conclusions:**

Our data demonstrate that BNT162b2 vaccinated individuals generate relevant nAbs titers, which begin to decrease within three months after immunization and show lower neutralizing potential against VOCs as compared to the wildtype strain. Large proportion of vaccine-induced S1-IgG might be non-neutralizing whereas RBD-IgGAM appears to be a good surrogate marker to estimate nAb levels. A third vaccination increases the nAb response. Furthermore, the systemic vaccine does not seem to elicit readily detectable mucosal immunity.

## Introduction

Starting from the pandemic spread of the coronavirus disease in December 2019 (COVID-19), global research efforts were made to identify effective vaccine candidates. Vaccines based on vectors, inactivated viruses and mRNA were licensed. The kinetics of SARS-CoV-2 antibody production and the persistence of humoral immunity in vaccinees are essential for national health services and the clinical management of the pandemic.

Studies suggested that SARS-CoV-2 specific antibody production following double vaccination with the BNT162b2 (BioNTech/Pfizer) mRNA vaccine is comparable to seroconversion following recovery from COVID-19 [1]. During SARS-CoV-2 infection, the median IgG seroconversion time has been estimated at 11 days post-symptom onset [2]. In patients with mild illness, specific subsets of antibodies, such as anti-nucleocapsid IgG, wane more rapidly, whereas antibodies against the viral spike protein including neutralizing antibodies (nAbs) remain detectable much longer [3–5]. To properly characterize the BNT162b2 vaccine and infection induced antibody response as well as to identify suitable diagnostic surrogate markers for difficult to determine nAbs, we analyzed and correlated different binding antibody assays to nAbs. We compared vaccine-derived to well characterized sera from COVID-19 convalescent patients of different disease severities. We also dissected the SARS-CoV-2 specific antibody production in individuals that were vaccinated with a heterologous ChAdOx1-S (AstraZeneca) prime and BNT162b2 (BioNTech/Pfizer) boost regime.

Furthermore, we performed a prospective study after the second BNT162b2 vaccination and assessed the levels of vaccine-induced antibodies three and eight months after the second vaccination dose as well as after a third vaccination.

Currently available vaccines were designed on the basis of the ancestral sequence of SARS-CoV-2 and have shown to elicit durable and potent immune responses to prevent from severe COVID-19 [6,7]. However, since the beginning of the pandemic several virus variants emerged globally with various amino acid substitutions, deletions or insertions in the viral proteins leading to enhanced transmissibility of the virus or immune escape effects [8–12]. To get insight into the neutralizing capacity of circulating antibodies elicited by the BNT162b2 vaccine against VOCs, we analyzed BNT162b2-induced nAbs after two and three doses against the variants of concern B.1.351 (Beta), B.1.617.2 (Delta) and B.1.1.529 (Omicron) [13–16].

In addition to systemic immunity, mucosal immune responses are considered to be critically important in reducing viral spread [17]. By mediation of the mucosa-associated lymphoid tissue (MALT), a strong suppression of SARS-CoV-2 transmission can be achieved due to the blockage of viral entry in mucosal cells of the oral cavity and pharynx [18]. In saliva of recovered COVID-19 patients, IgA antibodies are detectable in high concentrations [19]. Due to their parental route of application, it remains unclear whether or not mRNA or heterologous vector/mRNA-combination vaccination regimes induce mucosal immune responses. To address this question we assessed SARS-CoV-2 specific IgA antibodies in saliva of vaccinees and convalescent COVID-19 patients.

## Patients and methods

### Study design and human samples

**Fig 1:**
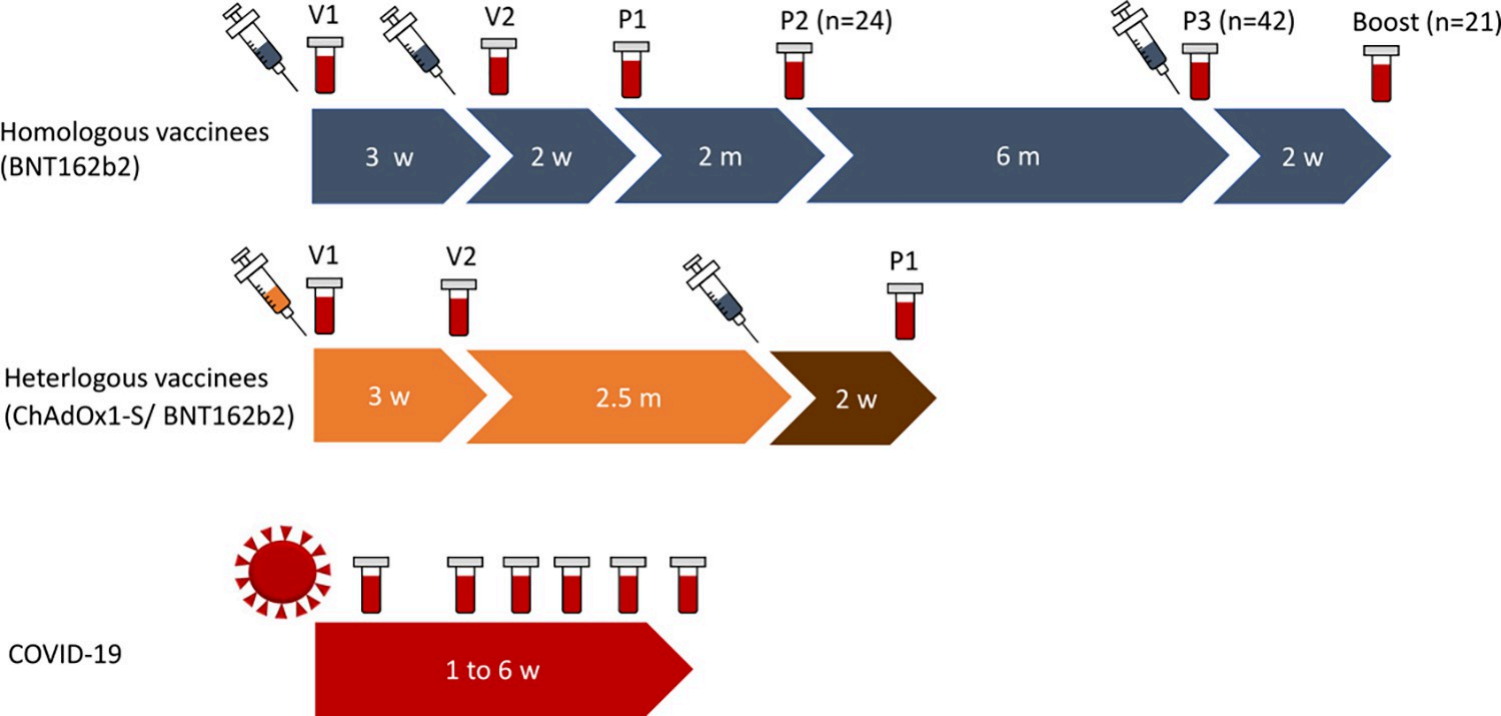
Time points of blood sampling within the different cohorts in median days Vaccinees: V1 prior first vaccination, V2 21 days after first vaccination, P1 21 days, P2 60 days and P3 240 days after second vaccination. Boost samples were taken 14 days after third vaccination. BNT162b2 vaccinees received their second vaccination 3 weeks after the first dose, whereas heterologous vaccinees received their second dose (BNT16b2) 10 weeks after the first vaccination with ChAdOx1-S. COVID-19 Patients: 35 days post symptom onset (PSO).

Two cohorts of vaccinated individuals and a set of bio-banked samples from COVID-19 patients were included into the study (Fig 1, Table 1). Study participants receiving vaccination with the BNT162b2 mRNA vaccine (BNT/BNT, n = 104, EMA fact sheet [20]) were recruited among healthcare employees at the Hospital St. Georg in Leipzig, Germany. Sera and saliva samples were collected on the day of first dose of vaccination (V1), on the day of the second dose vaccination (V2, median 21 days [IQR 21–22] after V1), and 14 to 28 days after the second dose vaccination (P1, median 42 days [IQR 42–43] after V1). Furthermore, additional serum samples were collected about 2.5 months after V2 (P2, 25 individuals, median 78 days [IQR 77–82], about 8 months after V2 (P3, 41 individuals, median 253 days [IQR 251–260] days after V2) and after a subsequent third vaccination (Boost) (20 individuals, median 15 days [IQR 14–16] days after P3).

**Table 1:** Demographic characteristics of vaccinees and COVID-19 cases.

	Homologous vaccinees (BNT/BNT)	Heterologous vaccinees (AZ/ BNT)	COVID-19 patients
N	104	11	57
Females (%)	68.3*	63.6*	43.9
Median age (min-max)	41 (20–66)	31* (26–37)	51 (32–79)
Median days after first dose (IQR) V2 P1 P2^1^ P3^2^ Boost^3^	21 (21–22) 42 (42–43) 102 (99–109) 276 (272–283) 288 (277–289)	21 (20–22) 91 (90–92) n/a n/a n/a	n/a
Median days after the onset of symptoms (min-max)	n/a	n/a	35 (7–43)
Vaccination reaction (%): None Local^4^ Systemic^5^	11.5 14.4 74.0	0 0 100	n/a n/a n/a
Severity of disease^6^ (%) mild (scale 2–3) moderate/severe (scale 4–5)	n/a n/a	n/a n/a	57.9 42.1

^1^ blood sampling was done for 25

^2^41, and

^3^20 of 104 volunteers.

^4^pain at the vaccination site.

^5^includes fever, chills, headache, fatigue, nausea, diarrhea, body aches, and nerve pain.

^6^Clinical severity of COVID-19 patients was classified according to the WHO clinical progression: (2) ambulatory without limitation of activity, (3) hospitalized without oxygen, (4) hospitalized on oxygen therapy by mask or nasal prongs, (5) hospitalized receiving non-invasive ventilation or high-flow oxygen therapy. Mann-Whitney-U test was used to analyze differences in age and Fisher’s exact test was applied to evaluate difference in gender

(* p<0.05, **p<0.01). d = days, IQR = inter quartile range, n.s. = not significant. AZ = AstraZeneca (ChAdOx1-S), BNT = BioNTech/Pfizer (BNT162b2), V2 = three weeks after first vaccination, P1 = two weeks after second vaccination, P2 = three month after first vaccination; P3 = eight months after first vaccination.

Another 11 study participants (63.6% females, median age 31 [IQR 26–37]) received a heterologous ChAdOx1-S vector based prime (EMA fact sheet [21]) and BNT162b2 boost vaccination (AZ/BNT). In accordance to the BNT/BNT group, serum samples were collected on the day of the first dose of ChAdOx1-S vaccination (V1), 21 days after V1 (V2, median 21 days [IQR 20–22] after V1), and 12 to 15 days after second dose vaccination (P1, median 91 days [IQR 90–92] after V1).

Inclusion into the study was independent of a previous SARS-CoV-2 infection. The ethics committee of the Saxonian medical chamber approved the study (registry number EK-allg-37/10–1) and informed written consent was obtained from all volunteers.

As reference, samples of 57 PCR-confirmed COVID-19 patients were included, which were treated between March 3 and November 11, 2020, at the Department of Infectious Diseases/Tropical Medicine, Nephrology and Rheumatology of the Hospital St. Georg. Serum samples were collected between 7 and 43 days (Median 35 days, IQR 20–44) after symptoms onset. COVID-19 patients were stratified into two groups according to the WHO clinical progression scale *(World Health Organization 2020 ordinal scale for clinical improvement)*: (1) “mild”, scale values 2 or 3 and (2) “moderate/severe” with scale values 4 or 5 [22]. For 29 of 34 COVID-19 patients with moderate/severe progression, saliva was collected (Table 1).

### Commercial assays for the detection of antibodies against S1 and viral nucleocapsid

All serum samples were tested for IgG against SARS-CoV-2 S1 (Anti-SARS-CoV-2-QuantiVac-ELISA, S1 Quant IgG; cut-off ≥25.6 BAU/ml) and for IgA against SARS-CoV-2 S1 (S1 IgA, Euroimmun, Lübeck, Germany; cut-off ratio ≥0.8). Samples above detection limit for S1 Quant IgG were pre-diluted 1:10 and 1:50 in sample buffer. In addition, baseline sera were screened for IgG antibodies against SARS-CoV-2 nucleocapsid (Virotech, Rüsselsheim, Germany; cut-off ≥11 VE/ml).

### In-house developed enzyme-linked immunosorbent assays

Detection of SARS-CoV-2 inactivated whole virion (IWV) IgG-antibodies and SARS-CoV-2 RBD polyvalent IgGAM-antibodies was performed according to Rockstroh *et al*. 2021 [3]. Briefly, Nunc PolySorp plates were coated with 1.5 μl per well of inactivated SARS-CoV-2 wt viral particles and 250 ng/well of RBD protein in 100 μl per well of carbonate coating buffer (15 mM Na2CO3, 7 mM NaHCO3 pH 9.6) overnight at 4°C. RBD protein (AA residues 329–538 of spike protein, strain Wuhan-Hu-1) was expressed in *Drosophila S2* cells and purified from cell culture supernatants with tandem immobilized metal affinity and size exclusion chromatography using the ÄKTA pure 25 l chromatography system (GeHealthcare). SARS-CoV-2 viral particles were purified from infectious cell culture supernatants by ultracentrifugation on a 30% sucrose cushion in MSE buffer (10 mM MOPS, pH 6.8, 150 mM NaCl, 1 mM EDTA) at 25,000 rpm for 3.5 h and 4°C. The pellet was resuspended in MSE buffer and chemically inactivated with 0.1% beta-propiolactone at 22°C. Sera (diluted 1:100) were incubated for 1.5 h at room temperature and binding antibodies were detected using a HRP-conjugated secondary goat anti human IgG antibody (Dianova, 1:20,000) or goat anti human IgG+IgM+IgA H&L antibody (Abcam, 1:10,000) for 1 h at room temperature. TMB substrate (Biozol) was added after a final wash step and incubated for 25 minutes before the reaction was stopped with 1 M H2SO4. Absorbance was detected at 450 nm with 520 nm as reference in a microplate reader (Tecan). The cut-off values were determined for each antigen individually and were validated using 100 pre-pandemic serum samples. All measurements were performed at least in duplicates.

### SARS-CoV-2 viral stocks

SARS-CoV-2 viruses were obtained from the European Virus Archive Global, EVAg (wildtype virus (wt) isolate BetaCoV/Germany/BavPat1/2020 and B.1.1.529 Omicron BA.1 or isolated from patient’s leftover samples (SARS-CoV-2 B.1.351 Beta isolate SARS-CoV-2/human/Germany/LE-B14HXA2/2021 and B.1.617.2 Delta isolate SARS-CoV-2/human/Germany/LE-B21AXB3/2021). Viruses were propagated in VeroE6 cells. Cells were grown to a confluence of approx. 80–90% and were infected at a MOI of 0.001 in Dulbecco’s modified Eagle’s medium (DMEM), supplemented with 2% FCS and 1% Pen/Strep. They were incubated for 48 h at 37°C with 5% CO_2_ until cytopathic effect (CPE) was visible. Virus containing supernatants were centrifuged at 4000 g for 10 min at 4°C and then stored at -80°C until use. Viral titers were determined using a focus-forming assay. All viral stocks were sequenced to verify their spike protein sequences and expected mutation sites.

### SARS-CoV-2 neutralization assay

Focus reduction neutralization assays (FRNT) were performed according to Rockstroh *et*. al. 2021 [3]. Briefly, heat-inactivated human serum samples were serially diluted in DMEM without FCS from 1:2.5 to 1:5120 and incubated with 50–150 focus forming units of SARS-CoV-2 wildtype, B.1.351, B.1.617.2 or B.1.1.529 for 1 h at 37°C before addition to confluent Vero E6 monolayers in 96-well plates. After an incubation of 1 h at 37°C, supernatant was removed, cells were washed with PBS, overlaid with 1% Methyl cellulose in DMEM with 2% FCS and incubated for 24–26 h at 37°C in 5% CO2. Cells were fixed with 4% paraformaldehyde in PBS, permeabilized and blocked with Perm-Wash buffer (0.1% saponin, 0.1% BSA in PBS). SARS-CoV-2 focus forming units were stained using a monoclonal rabbit anti-S1 antibody (CR3022, abcam, 1:1,000) and a secondary goat anti-rabbit IgG HRP-conjugated antibody (Dianova, 1:1,000). After the addition of TrueBlue substrate (Seracare), spots were counted with an ELISpot reader (AID Diagnostika). FRNT_90_ titers were determined as the reciprocal of the last dilution providing a minimum of 90% neutralization of focus forming units in comparison to the virus control without serum. A positivity cut-off of FRNT_90_ ≥5 was determined with negative reference sera.

### Collection and detection of SARS-CoV-2 specific IgA in saliva

Saliva was collected using the Oracol Saliva Collection System (Oracol, Worcester, U.K.) according to the manufacturer’s specification. Aliquots were stored at -80°C in Protein Low-Bind microtubes (Eppendorf, Hamburg, Germany) until further use. Samples were centrifuged at 10,000 g for 5 min and the supernatant was collected. 25 μl of saliva was mixed with equal amounts of LEGENDplex assay buffer and S1- or RBD-coated beads in a 5 ml polypropylene FACS tube, sealed and incubated overnight at 7°C in the dark. Subsequently, the bead mixture was washed with 1 ml of LEGENDplex wash buffer at 250 g for 5 min and incubated with 25μl of Streptavidin-conjugated anti-IgA detection antibody for 60 min on an orbital shaker at 800 rpm, followed by the addition of 25 μl of Streptavidin-PE conjugate for another 30 min (all from BioLegend, SanDiego, CA, U.S.). After another wash step, beads were resuspended in 500 μl of BD sheath fluid and analyzed using a BD FACS Lyric flow cytometer (BD Biosciences, San Jose, CA, U.S.) with PMT voltage settings adapted to discriminate beads specific for Spike S1- and Spike RBD-specific IgA antibodies. Binding of IgA antibodies was evaluated as median fluorescence signals on detected beads.

### Statistical analysis

SPSS version 21 (IBM, Armonk, NY, USA) and GraphPad PRISM version 6 (GraphPad Software, San Diego, CA, USA) were used for statistical calculations and generation of figures. Data was tested for gaussian distribution with Shapiro-Wilk normality test. Statistical tests for normally distributed values were calculated as paired or unpaired one-way ANOVA with Holm-Sidak’s multiple comparison test for the analysis of follow-up samples or vaccine and infection induced antibodies, respectively. For neutralization antibody titers, where Gaussian distribution was not confirmed, paired or non-paired Kruskal-Wallis with Dunn’s multiple comparison test to compare multiple timepoints or Wilcoxon matched-pairs signed rank test to compare two parameters was applied. Fisher’s exact test was applied for comparison of categorical variables.

## Results

### Serological characterization of BNT162b2 vaccinees and COVID-19 convalescents

**Fig 2:**
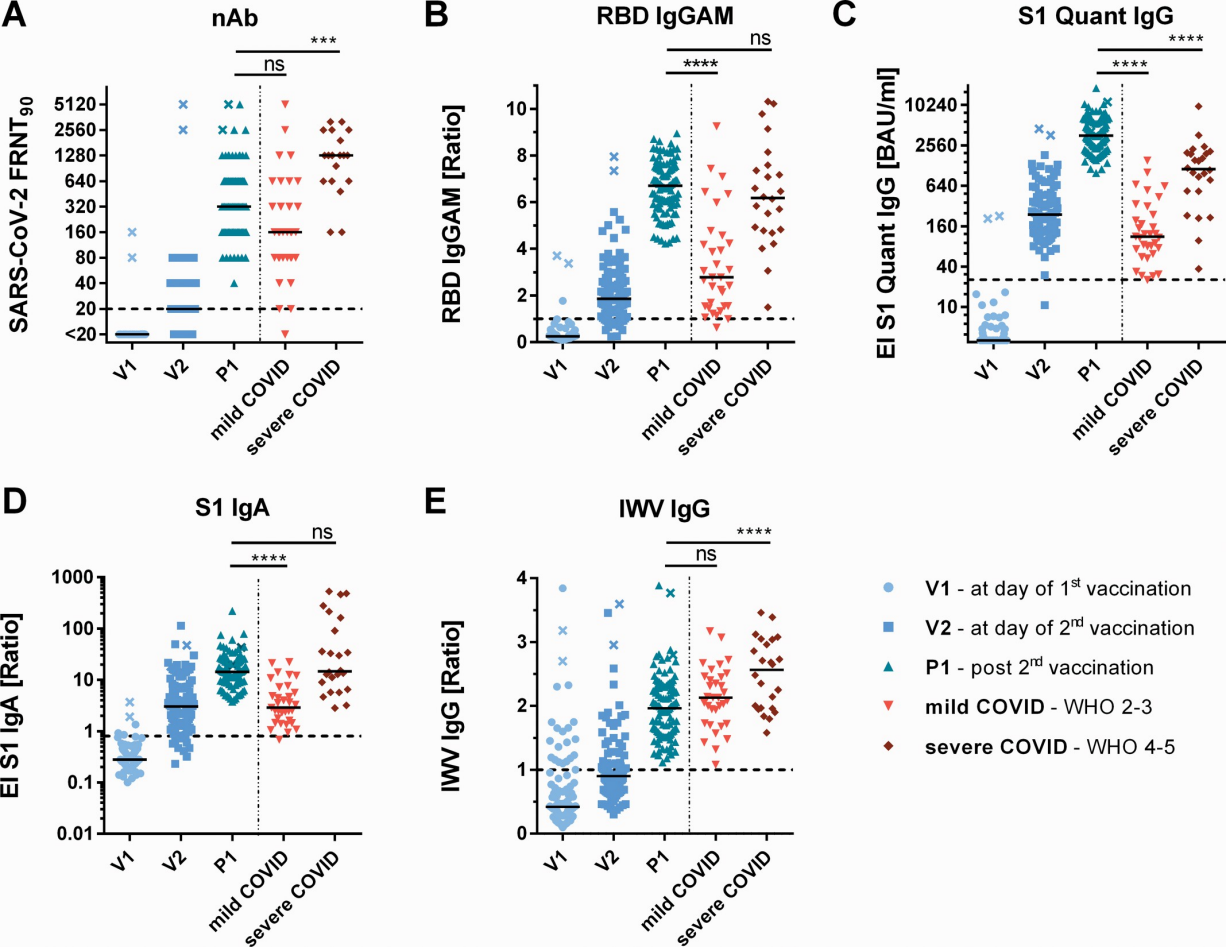
BNT162b2 vaccination induced antibodies in comparison to SARS-CoV-2 infection induced antibodies The presented data shows antibody ratios before first (V1), 3 weeks after first (V2), and 2 weeks after second vaccination (P1), as well as antibodies in sera of patients after mild and severe COVID-19 course. (**A**) Reciprocal titers of SARS-CoV-2 neutralizing antibodies were measured in focus reduction neutralization assay with 90% inhibition (FRNT_90_). (**B**) RBD IgGAM signals determined as sample/cut-off ratios (**C**) S1 quant IgG antibodies were quantitatively measured in binding antibody units per milliliter (BAU/ml). (**D-E**) S1-IgA and inactivated SARS-CoV-2 whole virus IgG signals (IWV) were determined as sample/calibrator ratios. The horizontal dotted lines represent positivity cut-offs. * = *p*<0.05, ** = *p*<0.01, *** = *p*<0.001, **** = *p*<0.0001, ns = not significant. x-marked data points represent vaccinees with a previous SARS-CoV-2 infection.

Among 104 individuals receiving a BNT162b2 vaccination we analyzed S1, RBD, inactivated whole virion (IWV) and neutralizing antibodies and drew comparisons to 57 COVID-19 convalescent individuals with mild or severe courses serving as reference group (Table 1, Fig 2A–2E) [3]. Three weeks after the first vaccination, SARS-CoV-2 neutralizing as well as S1, RBD and IWV binding antibodies were detectable in most individuals. Two weeks after the second dose, nAb titers and binding antibodies to all tested antigens were significantly increased. At this time point, nAb titers (Median FRNT_90_ = 320) as well as IWV IgG antibody signals in vaccinees were comparable to those induced by mild COVID-19 but did not reach the high levels of patients having severe COVID-19 courses (4- and 1.3- fold decrease for nAb and IWV IgG respectively) (Fig 2A and 2E). In contrast, vaccine-induced RBD IgGAM and S1 IgA serum antibody levels corresponded to those induced by severe COVID-19 and were even significantly exceeded by vaccine-induced S1 IgG antibodies (3.5-fold increase). For some individuals, IgG antibodies against the inactivated whole virion of SARS-CoV-2 (IWV) were detected even before the first vaccination. In comparison to BNT162b2 vaccinees, the heterologous AZ/BNT vaccination cohort showed similar nAb titers after the first dose (S1 Fig). However, after the second vaccination their nAb titers (4-fold increase) were significantly higher than in the mRNA vaccine group.

**Fig 3:**
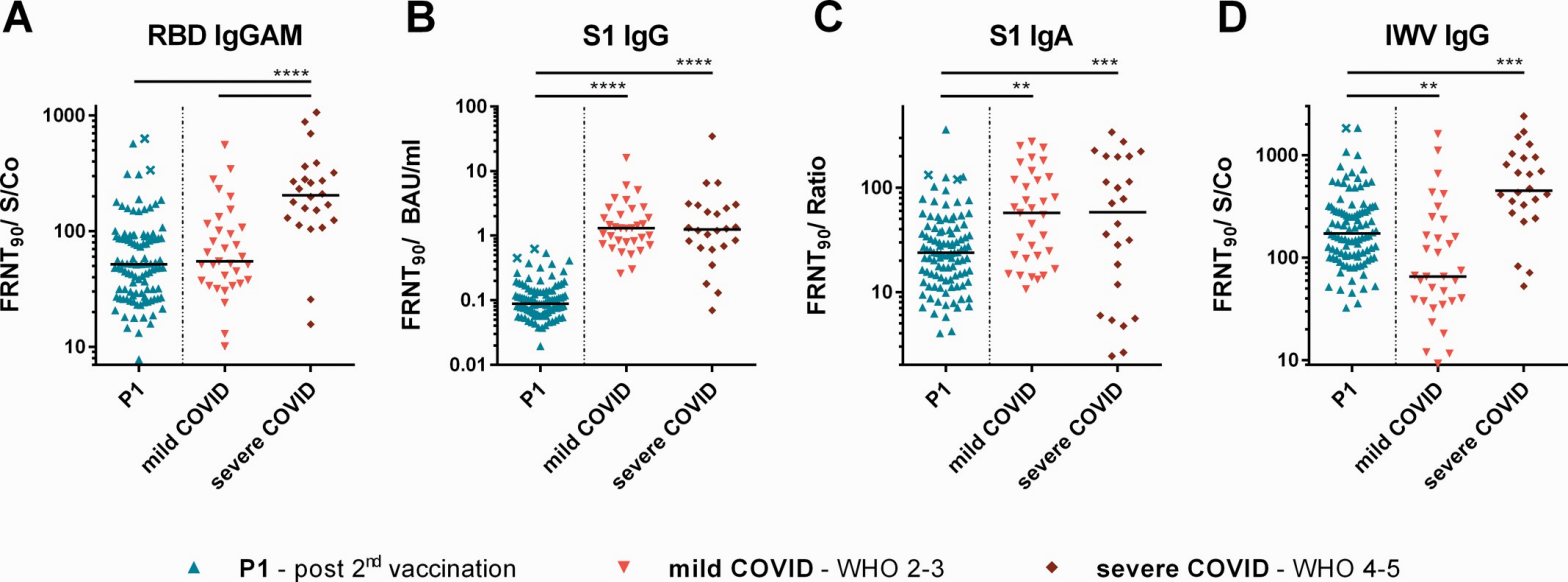
Ratios between SARS-CoV-2 neutralizing antibody titers and (**A, C, D**) RBD IgGAM, S1 IgA, IWV in sample/calibrator ratios and (**B**) S1 quant IgG in BAU/ml. Sera of vaccinees two weeks after second vaccination (P1) were compared with sera of COVID-19 patients with mild and severe courses according to WHO score (2–3 mild and 4–5 severe). * = *p*<0.05, ** = *p*<0.01, *** = *p*<0.001, **** = *p*<0.0001, ns = not significant.

The correlation between BNT162b2-induced S1 IgG, RBD IgGAM antibody signals and nAb titers using Spearman’s rank coefficient is presented in S2 Fig. Herein, S1 IgG and RBD IgGAM antibodies indicated the strongest correlation to SARS-CoV-2 nAbs (*r* = 0.93) (Fig 3A and 3B), whereas IWV and S1-IgA correlation coefficients ranged between *r* = 0.722 and 0.817 (S1C and S1D Fig).

### Comparison of binding and neutralizing SARS-CoV-2 antibodies

Ratios of binding to neutralizing antibodies were calculated to compare the proportion of neutralizing antibodies induced by vaccination to convalescent individuals in each test (Fig 3). Vaccinees presented a significantly lower proportion of neutralizing to S1 binding antibodies in comparison to the COVID-19 group (10- fold and 5-fold lower for S1 IgG and S1 IgA respectively). Similarly, this ratio was lower for RBD binding IgGAM antibodies compared to patients after severe COVID-19 but was found comparable to the mild COVID-19 group. For IWV-IgG the neutralizing proportion of binding antibodies was higher than in patients with mild COVID-19 courses but below the median of severe courses.

### Long term kinetics of antibody titers

**Fig 4:**
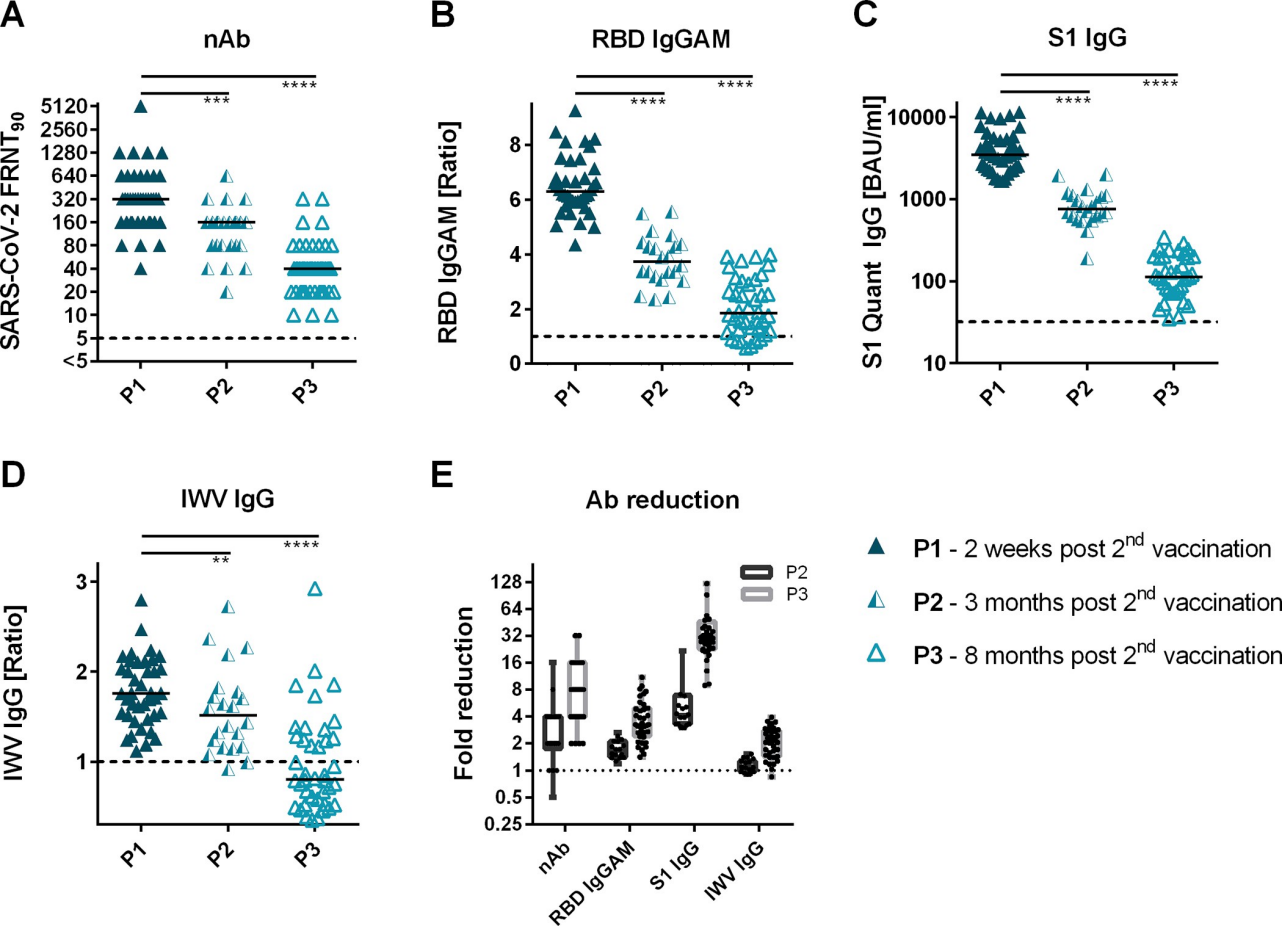
BNT162b2-induced antibody levels 2 weeks, 3 and 8 months after first vaccination. (A) Reciprocal titers of SARS-CoV-2 neutralizing antibodies were measured in focus reduction neutralization assay with inhibitor titer 90% (FRNT90). (B) RBD IgGAM signals were determined as sample/calibrator ratios (C) S1 IgG antibodies were quantitatively measured in binding antibody units per milliliter (BAU/ml). (D-E) S1 IgA and IWV (SARS-CoV-2 inactivated whole-virion) titers were determined as sample/calibrator ratios. (F) Fold reduction of SARS-CoV-2 neutralizing antibody titers, RBD IgGAM, S1 IgG, S1 IgA and IWV IgG antibody signals in follow-up vaccine sera calculated as ratio of value on timepoint P1 to timepoint P3.

The kinetics of antibody abundance varied greatly between individuals, with a mean titer reduction of nAbs by 3.3-fold at P2 and 9.7-fold at timepoint P3 compared to P1 two weeks after second vaccination (Fig 4A). RBD IgGAM ratios decreased 2.1-fold after three months and 4-fold after eight months (Fig 4C). S1 antibody concentrations reduced 5.7-fold at timepoint P2 and 36.6-fold at P3 (Fig 4C). Smallest differences were observed in the IWV IgG antibody signals with a 1.1-fold mean reduction after three months and a 2.2-fold mean reduction after eight months (Fig 4D).

### Neutralizing effect of BNT162b2-induced antibodies on Beta, Delta and Omicron VOCs

**Fig 5:**
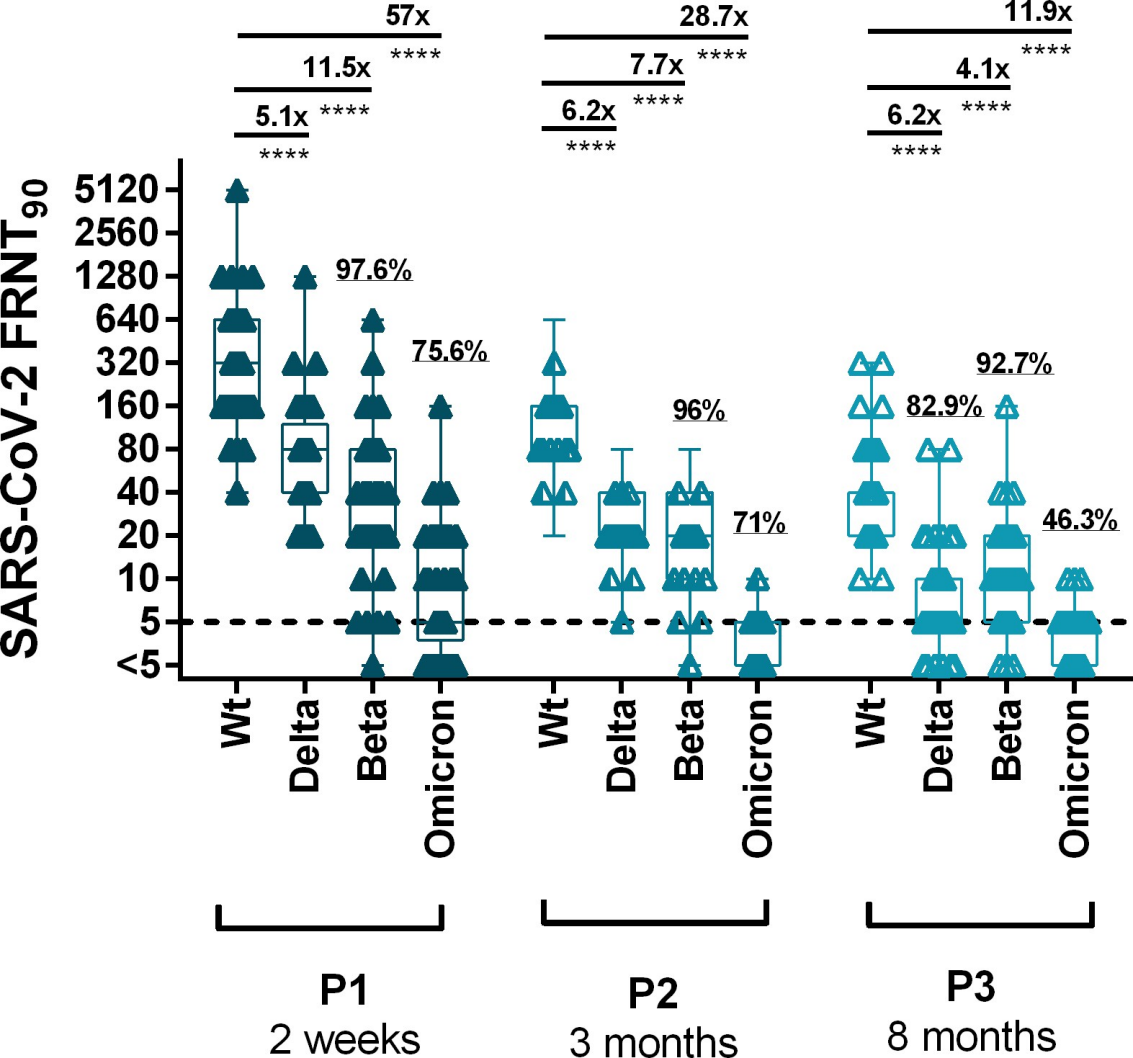
BNT162b2- and infection induced reciprocal SARS-CoV-2 nAb titers on wildtype strain wt-BavPat1 and VOC strains Delta, Beta and Omicron at timepoint P1 to timepoint P3. The dotted line indicates the limit-of-detection at a titer of 1:5. FRNT90: Focus reduction neutralization titer at 90% virus inhibition; results plotted as reciprocal values. Proportion of individuals with remaining nAbs in percent. Mean neutralizing titer reductions of SARS-CoV-2 wt to VOC-nAb are depicted above the continuous lines * = *p*<0.05, ** = *p*<0.01, *** = *p*<0.001, **** = *p*<0.0001, ns = not significant.

Neutralizing antibody titers were decreased towards VOCs in almost all tested individuals and cohorts when compared with wildtype SARS-CoV-2 (Fig 5). Two weeks after the second vaccination, mean nAb titers decreased by 5.1-, 11.5- and 57-fold with the Delta, Beta, and Omicron variant, respectively. With 6.2-fold and 7.7-fold a similar nAb mean reduction was observed for Delta and Beta variants at P2. For Omicron a 28.7-fold reduction was observed at time point P2. After eight months nAb titers for Delta showed a 6.2-fold, for Beta a 4.1-fold and for Omicron a 11.9-fold decrease. Compared to the homologous group vaccinated only with mRNA, the decrease of nAbs against Beta and Omicron was lower (3.5- and 23-fold decrease, respectively) in the heterologous AZ/BNT vaccination cohort. For Delta the AZ/BNT group showed a similar reduction (5.8-fold reduction) as the group of mRNA vaccinated individuals (S3 Fig).

### Impact of a third vaccination on nAb titer level on wildtype, Beta, Delta and Omicron strains

**Fig 6:**
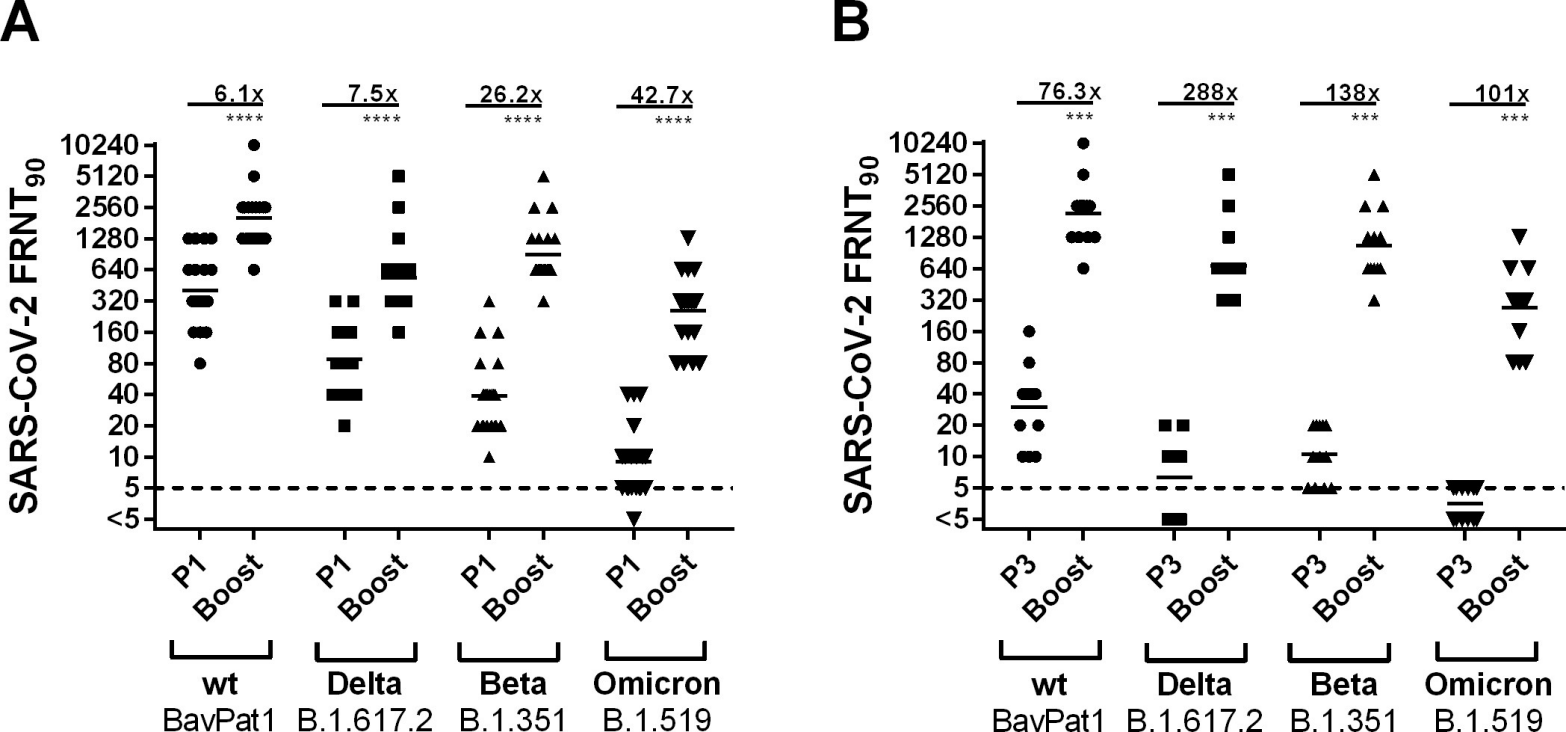
SARS-CoV-2 nAb titers on the wildtype strain wt-BavPat1 and the VOC strains Delta, Beta and Omicron after third vaccination (Boost) (A) compared to P1 two weeks after second vaccination and (B) compared to P3 8 months after first and third vaccination (Boost). Only individuals that had provided samples at both time points were compared. The dotted line indicates the limit-of-detection at a titer of 1:5. FRNT90: Focus reduction neutralization titer at 90% virus inhibition; results plotted as reciprocal values. Mean neutralizing titer reductions are depicted above the continuous lines * = *p*<0.05, ** = *p*<0.01, *** = *p*<0.001, **** = *p*<0.0001, ns = not significant.

Two weeks after the third vaccination (Boost) nAb titers were significantly increased compared to two weeks after the second vaccination (P1). The median titer increase ranged from 6.1-fold for SARS-CoV-2 wildtype to 42.7-fold for Omicron (Fig 6A). Compared to P3 eight months after the second vaccination, median nAb titers after third vaccination were increased from 76.3- to 288-fold for wildtype and Delta, respectively (Fig 6B).

### Detection of SARS-CoV-2 targeting antibodies in saliva samples

**Fig 7:**
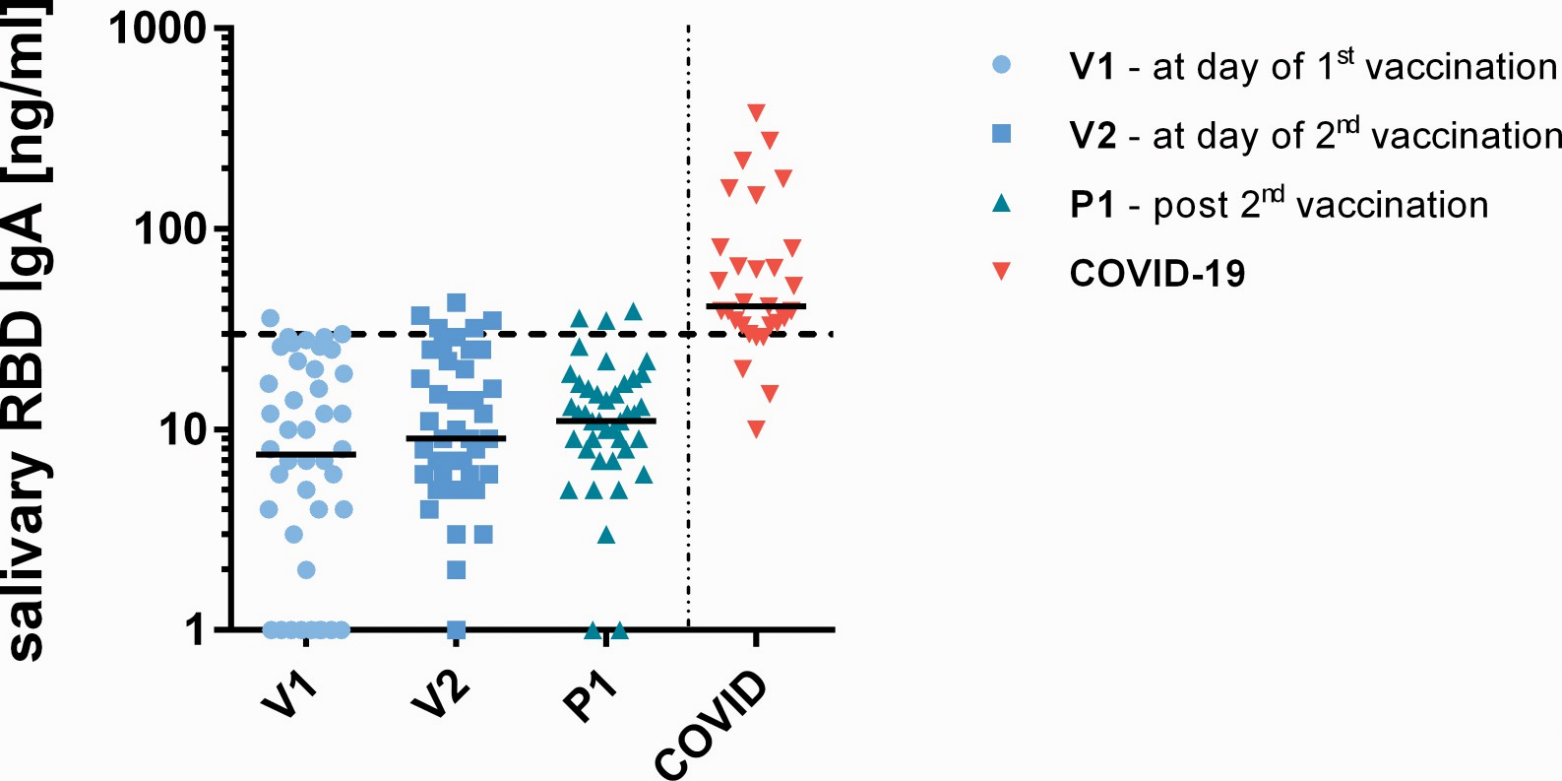
Salivary IgA specific to SARS-CoV-2 RBD in N = 40 individuals prior first (V1), three weeks after first (V2) and to weeks after second vaccination (P1) in comparison with COVID-19 patients (N = 31). The dotted line indicates the 95% interquartile range for vaccinees.

Saliva samples from vaccinees and COVID-19 patients with moderate or severe courses were assessed for S1- and RBD-specific IgA antibodies using a sensitivity-trimmed bead-based flow-cytometric assay. Based on interquartile range calculation, no increase of IgA production at V2 or P1 was observed in the group of BNT162b2-vaccinated individuals. In contrast, most COVID-19 patients had detectable salivary IgA towards SARS-CoV-2 antigens after 15–30 days after the onset of symptoms (Fig 7).

## Discussion

### Impact of vaccination against SARS-CoV-2 on antibody formation

Vaccination with mRNA- or vector-based vaccines represents a milestone in combating the SARS-CoV-2 pandemic. To better understand their protective effects, we assessed the antibody formation quantitatively and functionally after vaccination in comparison with SARS-CoV-2 infections. Three weeks after the first vaccination with BNT162b2, in half of the subjects nAbs could be detected, which is of note with regard to recent studies linking early levels of nAbs with protection against SARS-CoV-2 [23,24]. This supports a presumably protective effect already after the first vaccination with the BNT162b2 mRNA vaccine, which previously has been demonstrated by a study with US health care personnel [25]. Two weeks after the second dose, all individuals developed high S1- and RBD-binding as well as SARS-CoV-2 nAb titers. These results are in concordance with similar observations in studies on mRNA vaccines [26–28]. Interestingly, an even stronger nAb production was observed in the group of heterologous ChAdOx1-S/BNT162b2 vaccinated individuals. This indicates that a combination of different SARS-CoV-2 vaccine classes can lead to similar or stronger humoral immune response which may result in a better protective effect, as has also been shown by other studies [29–33].

### Kinetics of SARS-CoV-2 antibody response after BNT162b2 vaccination

We have observed significant reduction of vaccine-induced antibody levels already after three months, which was even more pronounced eight months after the first vaccination. Interestingly, S1 quant IgG and IgA antibodies decreased strongly, whereas nAb levels and RBD-IgGAM dropped to a lesser extent [34]. Thus, the S1 quant IgG ELISA does not seem to be an optimal diagnostic choice for determining longevity of humoral immune responses after SARS-CoV-2 mRNA vaccination. This is at least the case for the test systems used here. The S1 IgG antibody kinetic might be different in assays of other manufactures [35]. Nevertheless according to our data, we propose the use of RBD-IgGAM determination as a rapid and simple surrogate marker to estimate the levels of nAbs after SARS-CoV-2 mRNA vaccination. One explanation for a better correlation between RBD-IgGAM and nAbs could be a better RBD-presentation in the vaccine antigen. This is supported by a study showing a broader nAb production against the RBD region in vaccinees compared to COVID-19 patients [36].

The significant decrease in SARS-CoV-2 antibody titers within a short diagnostic interval strengthens the demand of a third vaccination dose from a serological point of view, which has already been supported by real world data [37,38]. However, the measurement of circulating antibodies upon vaccination or infection does not deliver the full picture of protection from disease. Instead, cellular and memory immune responses have to be taken into account, as well. Data from recovered COVID-19 patients implies the presence of memory B- and T-cells in almost all individuals up to eight months after SARS-CoV-2 infection [5,39].

### Vaccination or SARS-CoV-2 infection induced nAbs against SARS-CoV-2 VOCs

The BNT162b2 vaccine was designed using the spike gene sequence of the ancestral Wuhan SARS-CoV-2 wildtype strain. During the pandemic spread of SARS-CoV-2, viral variants emerged with various mutations, leading to amino acid substitutions, deletions or insertions in the spike protein. These provide potential for increased transmissibility of the virus [40,41]. Compared to wildtype strain our data revealed a significant but only moderate reduction in the effectiveness of neutralizing antibodies against the B.1.617.2 (Delta) and B.1.351 (Beta) VOC two weeks after second vaccination, thereby providing further evidence for mRNA vaccination efficacy against this variants [42–45]. A substantial (57-fold) reduction was observed with the B.1.1.529 (Omicron) variant with a few individuals showing only poor or no neutralizing response at all. These results are in line with recent findings of other serological studies and real world data which indicate that two vaccine doses provide only a very limited neutralizing antibody activity or protection from symptomatic disease against the Omicron variant [46–50].

Data of individuals that were vaccinated with ChAdOx1-S and subsequently with BNT162b2 showed a lesser reduction of nAbs targeting VOCs after two vaccinations compared to the group vaccinated exclusively with mRNA. This leads to the conclusion that the combination of both vaccines results in more robust immune response regarding VOC infections. However, the group size for the heterologous vaccination is rather small compared with the mRNA vaccinated one, but our results support recent findings of a large cohort study [33].

The third immunization led to a significantly stronger production of SARS-CoV-2 nAbs compared to the second vaccination as it was expected based on previous findings [51]. Of note, the mean increase was even more pronounced for SARS-CoV-2 variants, which indicates that a third vaccine dose not only raises the antibody titers but also enhances the quality of the produced antibodies through maturation and antibody avidity as also observed in other studies [52–54]. This finding is relevant for antibody-mediated protection against the Omicron variant. Here, a neutralizing immune response was now detected in all boosted individuals.

### BNT162b2 vaccination and mucosal immune response

Generation of an intermittent protective mucosal immunity is generally accepted in COVID-19 patients undergoing natural infection and constitutes a relevant component in the suppression of pandemic SARS-CoV-2 dissemination. However, using available COVID-19 mRNA and vector-based vaccines, an immune response of mucosa associated lymphoid tissue (MALT) is highly questionable. The protection of most systemic vaccinations against mucosal infection solely relies on few circulating IgA and IgG antibodies which transudate from sera into the mucosa [55]. We have not found detectable antibodies against SARS-CoV-2 in the saliva using our methods. These results suggest that SARS-CoV-2 mRNA vaccination is not able to trigger a detectable protective mucosal immune response. However, other groups recently detected S1-IgA, IgG and RBD IgG antibodies IgG antibodies in saliva of vaccinated individuals [56,57]. One explanation for the apparently discrepant results might be, that the detected IgA antibodies in these studies are most likely transudated plasma S1 antibodies. Our data suggest that the production of circulating IgA might be weaker against the RBD than against the full length S1 subunit. In order to increase mucosal immunity, development of mucosal vaccines could be crucial to critically interfere with the pandemic spread of SARS-CoV-2.

### Conclusions

BNT162b2 mRNA vaccination provided sustainable formation of SARS-CoV-2 neutralizing antibodies in the present study cohort. To induce a detectable neutralizing immune response against the Omicron VOC and to preserve detectable nAb titers in serum against other SARS-CoV-2 variants during 6 months, a third vaccination seems to be necessary. Regarding mucosal immune response, the present analysis showed no relevant induction of salivary IgA after mRNA vaccination.

## Supporting information

S1 Fig BNT162b2-induced antibodies and SARS-CoV-2 virus neutralizing titers. (PDF)

S2 Fig Comparison of antibody titers between BNT162b2 and ChAdOx1-S/BNT162b2 vaccinated individuals. (PDF)

S3 Fig Neutralizing antibodies in ChAdOx1-S/BNT162b2 vaccinated individuals to SARS-CoV-2 wild-type and VOCs. (PDF)
