# Supplementary material for: Evaluation of the systemic and mucosal immune response induced by COVID-19 and the BNT162b2 mRNA vaccine for SARS-CoV-2
Source: PLoS One. 2022 Oct 18;17(10):e0263861. doi: 10.1371/journal.pone.0263861 (PMC9578597; doi:10.1371/journal.pone.0263861)
Supplement: S1 Fig — (PDF) [file pone.0263861.s001.pdf]

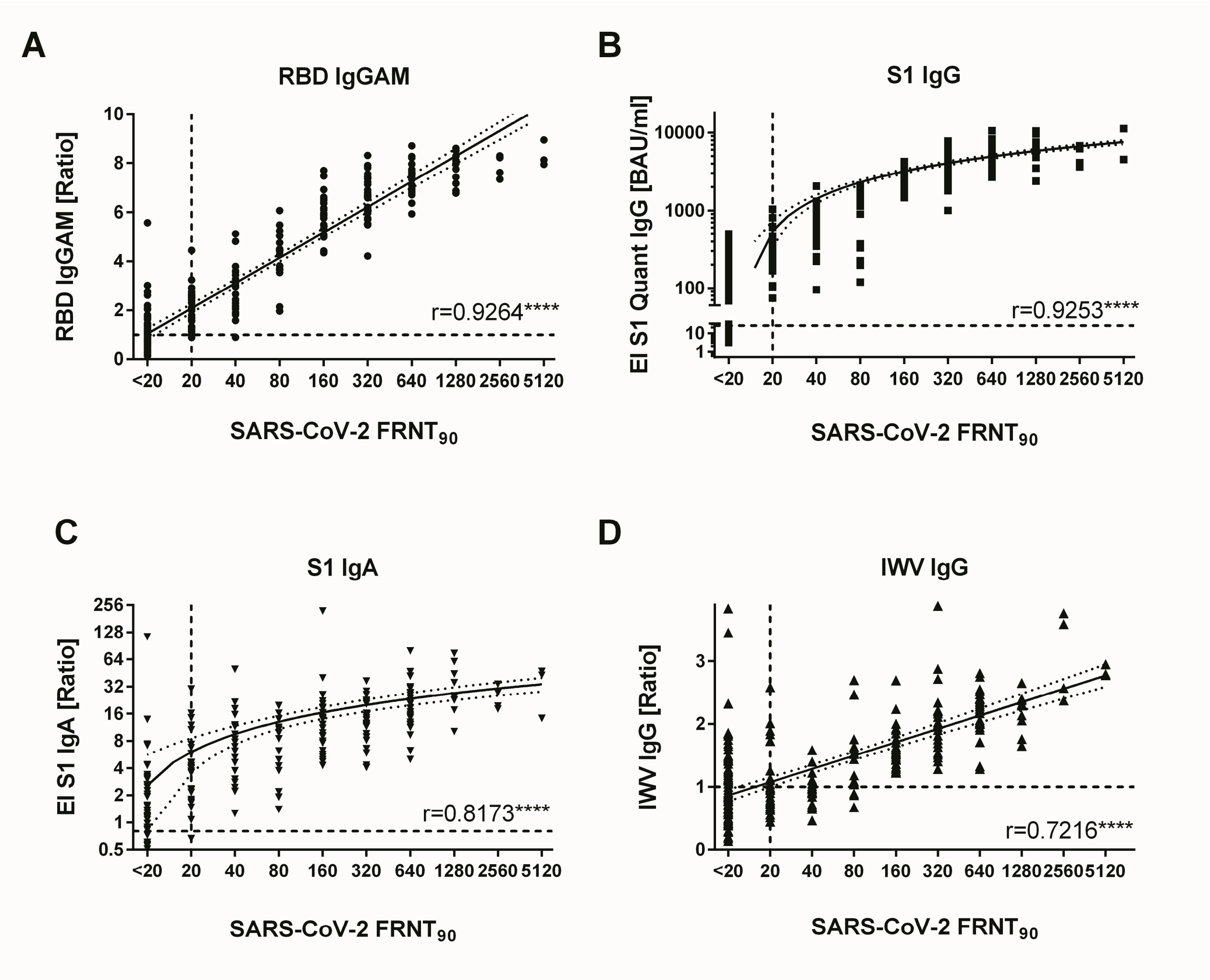

S1 Fig: Correlation between different BNT162b2-induced antibodies and SARS-CoV-2 virus neutralizing titers Horizontal dotted line represents the cut-off values of the ELISAs. The vertical dotted lines show the neutralization assay cut-off. (**A**) RBD IgGAM as sample/calibrator ratio (**B**) S1 IgG antibodies were measured quantitatively [BAU/ml] (**C-D**) IWV IgG and S1 IgA antibodies are depicted as sample/calibrator ratios.
