# Supplementary material for: Evaluation of the systemic and mucosal immune response induced by COVID-19 and the BNT162b2 mRNA vaccine for SARS-CoV-2
Source: PLoS One. 2022 Oct 18;17(10):e0263861. doi: 10.1371/journal.pone.0263861 (PMC9578597; doi:10.1371/journal.pone.0263861)
Supplement: S2 Fig — (PDF) [file pone.0263861.s002.pdf]

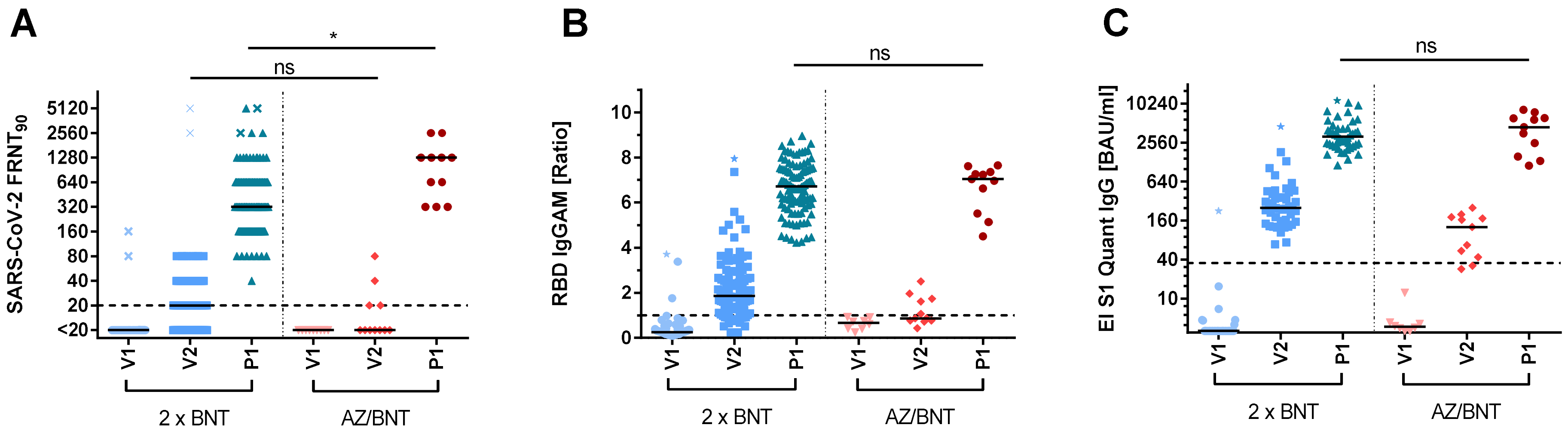

S2 Fig: The presented data compare antibody titers between homologous BNT162b2 (BNT) and heterologous ChAdOx1-S/BNT162b2 (AZ/BNT) vaccinated individuals at time points before first (V1), three weeks after the first (V2), and two weeks after the second vaccination (P1). (**A**) Titers of neutralizing antibodies (nAb) were measured in focus reduction neutralization assay with inhibitor titer 90% (FRNT90). (**B**) RBD IgGAM signals determined as sample/cut-off ratios (**C**) S1 quant IgG antibodies were quantitatively measured in binding antibody units per milliliter (BAU/ml).

The horizontal dotted lines represent the positivity cut-offs for each test. Statistical analysis was performed with ordinary 1-way ANOVA, Holm-Sidak's multiple comparison test * = p<0.05, ** = p<0.01, *** = p<0.001, **** = p<0.0001, ns = not significant
