# Supplementary material for: Evaluation of the systemic and mucosal immune response induced by COVID-19 and the BNT162b2 mRNA vaccine for SARS-CoV-2
Source: PLoS One. 2022 Oct 18;17(10):e0263861. doi: 10.1371/journal.pone.0263861 (PMC9578597; doi:10.1371/journal.pone.0263861)
Supplement: S3 Fig — (PDF) [file pone.0263861.s003.pdf]

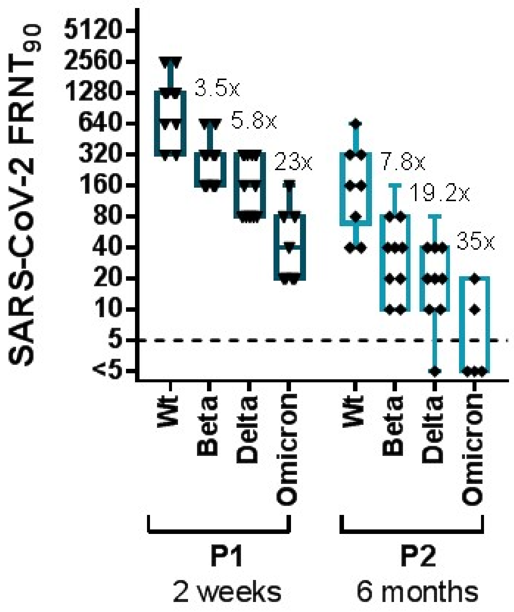

S3 Fig: Vaccine induced neutralizing antibodies of heterologous ChAdOx1-S/BNT162b2 (AZ/BNT) vaccinated individuals to SARS-CoV-2 wild-type (wt-BavPat1) and SARS-CoV-2 VOCs Beta, Delta and Omicron.The dotted line indicates the limit-of-detection at a FRNT90 titer of 1:5. The results were plotted as reciprocal titers. Statistical analysis was performed with ordinary 1-way ANOVA.
